# Identification of hot regions in hub protein–protein interactions by clustering and PPRA optimization

**DOI:** 10.1186/s12911-020-01350-4

**Published:** 2021-05-03

**Authors:** Xiaoli Lin, Xiaolong Zhang

**Affiliations:** Hubei Key Laboratory of Intelligent Information Processing and Real-Time Industrial System, School of Computer Science and Technology, Wuhan University of Science and Technology, Wuhan, 430065 People’s Republic of China

**Keywords:** Hub protein, Protein–protein interactions, Optimization, Clustering

## Abstract

**Background:**

Protein–protein interactions (PPIs) are the core of protein function, which provide an effective means to understand the function at cell level. Identification of PPIs is the crucial foundation of predicting drug-target interactions. Although traditional biological experiments of identifying PPIs are becoming available, these experiments remain to be extremely time-consuming and expensive. Therefore, various computational models have been introduced to identify PPIs. In protein-protein interaction network (PPIN), Hub protein, as a highly connected node, can coordinate PPIs and play biological functions. Detecting hot regions on Hub protein interaction interfaces is an issue worthy of discussing.

**Methods:**

Two clustering methods, LCSD and RCNOIK are used to detect the hot regions on Hub protein interaction interfaces in this paper. In order to improve the efficiency of K-means clustering algorithm, the best k value is selected by calculating the distance square sum and the average silhouette coefficients. Then, the optimization of residue coordination number strategy is used to calculate the average coordination number. In addition, the pair potentials and relative ASA (PPRA) strategy is also used to optimize the predicted results.

**Results:**

DataHub dataset and PartyHub dataset were used to train two clustering models respectively. Experiments show that LCSD and RCNOIK have the same coverage with Hub protein datasets, and RCNOIK is slightly higher than LCSD in Precision. The predicted hot regions are closer to the standard hot regions.

**Conclusions:**

This paper optimizes two clustering methods based on PPRA strategy. Compared our methods for hot regions prediction against the well-known approaches, our improved methods have the higher reliability and are effective for predicting hot regions on Hub protein interaction interfaces.

## Background

Proteins perform their various biological functions by interacting with other proteins, nucleic acids or small molecules [1, 2]. Recognizing the interaction of proteins can reveal the mechanism of protein activities and promote the development of biotechnology and life science [3, 4]. Studying the function and structure of proteins is very important for understanding various life activities [5, 6]. At present, the functions of many proteins are still unclear. Most molecular and cellular processes are controlled by PPIs. Understanding the mechanism of PPIs is essential for understanding large-scale tissues of cells and their biological pathways.

For many years, a lot of new technologies have emerged to identify PPIs [7–9]. These technologies have been used to construct large-scale cellular networks for many organisms, which are composed of interacting proteins to achieve specific biological functions. Graph theory is used to analyze these networks to promote understanding of cellular functions and tissues [10]. The most important discovery is that there is a close relationship between the network’s topological characteristics and the basic functions, that is, the number of interactions between proteins is related to the importance of their functions.

Early studies on the interaction network of yeast protein revealed that PPIN belongs to the scale-free topology, with a small number of highly connected proteins (called Hub proteins) and a large number of low-connectivity proteins [11]. In these networks, genes or proteins are defined as nodes, and their interactions are defined as edges. The importance of nodes can be measured according to the changes in network functions or organism adaptability caused by removing the node. Genome-wide studies have shown that a small number of genes in the genome are indispensable for survival. Researches showed that Hub protein is essential in the scale-free PPIN. The loss of Hub protein may be more fatal than that of non-Hub protein. This phenomenon is called the central-lethal rule, which reflects the special importance of Hub protein in the network and shows the biological significance of network architecture. Studying PPIs provides basic ideas for understanding the properties and interactions of different Hub protein complexes [12]. Hub proteins with high connectivity are highly conserved proteins, which participate in the processes of signal transduction [13, 14]. The functions of some conserved Hub proteins are unknown. The in-depth understanding of the functions of Hub proteins in the interaction network will help to regulate and interfere with protein interactions. Hub protein as a drug target can be used to design drugs and treat diseases [15]. For example, in cancer research, Hub protein with high expression in diseased tissues may be a target.

Over the years, many researchers have studied the relationship between hub protein and topological centrality in PPIN. Jeong et al. [11] studied Saccharomyces cerevisiae PPIN and found that the network shows power-law connectivity distribution. Yu et al. [16] found that proteins with high marginal importance tend to be Hub proteins, which have more interactions and shorter characteristic path lengths than other proteins. Hahn et al. [17] pointed out that most proteins do not evolve in isolation. So, the location of proteins in the network can indicate their centrality to cellular function. Batada et al. [18] showed that Hub protein has at least one unusual set of characteristics, such as high decay rate of mRNA and a large number of phosphorylation sites, and Hub protein is more important for cell growth rate. He et al. [19] found the relationship between the structural importance of Hub node and its functional importance in PPIN, and reveal the biological significance of network structure. Therefore, it is necessary to carry out research on the Hub protein interaction network, which has important theoretical guiding for drug design.

In PPIs, the hot spot residues [20, 21] are usually surrounded by other non-hot spot residues, and the filling density of hot spot residues is much higher than that of non-hot spot residues. In PPIs, the hot spots form a specific conformation, called the hot region [22]. Cukuroglu et al. [23] pointed out that PartyHub and DateHub are different in function and evolution. They also analyzed the hot region organizations of Hub proteins, and found that different protein partners can bind to hot spots of different types of hub protein interface. This paper mainly discusses hot regions on the interfaces of Hub protein.

The essence of hot region identification is to find out the most stable structural conformation in the interface. Its physical properties determine the spatial layout of residues, including spatial potential energy and spatial distance. And its biological properties determine the pairing tendency between residues. Therefore, the calculation method can be used to simulate the biological structure of hot region more comprehensively, and then the machine learning algorithm can be used to speed up performance. Therefore, combining the three-dimensional structure of proteins and the machine learning technology to analyze the physicochemical characteristics of protein residues, some works have been carried out to detect the hot regions in PPIs [22]. The hot region in PPIs is just like the community in the complex network. And the community is a hidden pattern in the complex network, which is also called the clustering of the complex network. So, in this paper, clustering method is used to detect the hot region structures in PPIs.

## Method

### Dataset

Because published data about hot spots of Hub proteins are fewer, we first used common hot spot datasets in published literature for better evaluating the performance of our methods. There are different definitions of hot spots, different datasets, and even different evaluation criteria in many researches. To more effectively evaluate our method, we first performed experiments used the same datasets as [24–26], which was obtained from the Alanine Thermodynamic Scanning Database (ASEdb).

Then, the Hub protein datasets were used to test, which were obtained from Ekman’s PPIN [27]. In this PPIN, the proteins are divided into PartyHub protein, DateHub protein and NonHub protein according to Ordered Locus Names (OLNs) and their Hub status. The features such as solvent accessible surface area (ASA) and relative accessible surface area (RASA) were obtained from HotPoint server. In addition, ASA values of monomers (RelMonomer ASA), ASA values of complexes (RelCompASA) and Potential Energy (Potential) were also obtained. The details of the datasets are shown in Table 1.

**Table 1:** Proportion of hot spots and non-hot spots in different datasets

Dataset	Hot spots	Non-hot spots	Total	Proportion
ASEdb	65	90	155	0.72:1
DateHub	1056	1850	2906	0.57:1
PartyHub	1033	1980	3013	0.52:1

**Table 2:** Selected optimal feature subset of protein residues

Rank	Feature	Rank	Feature
1	BsRASA	12	BbASA
2	BsASA	13	BbRASA
3	BsmDI	14	UbASA
4	BnRASA	15	UbRASA
5	BminPI	16	UsASA
6	BpRASA	17	UnASA
7	BmaxPI	18	UnRASA
8	BpASA	19	UtASA
9	BnASA	20	UsmDI
10	BtmDI	21	UmaxDI
11	BmaxDI	22	UminPI

### Feature selection

When the number of features exceeds a threshold for the limited training set, the accuracy will decrease with the increase of features to result in over-fitting. In addition, the number of features is proportional to training time of the model. Redundancy features will take more training time, and the most important is to reduce generalization ability on datasets. Therefore, the appropriate feature subset is help to construct the training model and can also increase the interpretability of the model. Protein residues have many chemical and physical characteristics. To effectively identify hot regions on Hub protein interaction interface, the feature selection method should be carried out to select the stable and optimum feature set. We have used several feature selection methods, such as the improved mRMR method [22] and SVM-RFE based on Pearson correlation coefficient [28]. Pearson correlation coefficient is an effective way to measure features. Using Pearson correlation coefficient, the features with high correlation can be found. To reduce redundancy, one of the highly correlated features can be randomly removed without much loss of information. So, this paper used the same method as [28] to select features. The selected optimal feature subset is shown in the Table 2.

### Algorithms

When predicting hot regions on the protein interface, more natural hot spots and fewer false hot spots (false positive) should be identified. Therefore, to obtain better prediction results, it is necessary to create a strong classifier for predicting hot spots. We have used some algorithms, such as gradient boosting [28] and random forest [21] to predict hot spots of the protein interaction interfaces. The number of residues in PPIN is numerous. The connections between residues are diverse and directional, and the network structures of residues have different characteristics. In addition, the residues in the network tend to packed closely together and form the different community structures. Within the community, the connections between residues are relatively dense and stable, while there are obvious differences between the different communities [29]. This inhomogeneous relationship indicates that there is a natural partition structure in PPIN. Choobdar et al.[30] also verified the existence of community structure in PPIs. Studies have shown that the hot region structure is independent in the protein interaction interface. Moreover, the relationship between hot spots in the same hot regions is strong, and the relationship between hot spots in different hot regions is weak. Hot spots and hot regions are defined as:

*Hot spot*: The residues with binding free energy $\ge 2.0$ kcal/mol are defined as hot spots [24]. The hot spots usually gather in the local tightly packed region. The hot spots in the same hot region are closely related to each other, and they have high coordination number.

*Hot region*: Supposing that each residue is regarded as a ball. If the distance between two hot spot residues is less than the sum of radius and a harmonic distance 2Å, the two hot spot residues are located in the same community. Three or more hot spots in the same community can form a hot region [5].

Hot spots tend to cluster tightly in a community, but they are unevenly distributed in the protein interaction interface. Therefore, hot spots can be clustered in a community by clustering method, and each cluster represents a set of similar objects. To effectively cluster hot spots, this paper used the PPRA optimization strategy to optimize LCSD algorithm (Local Community Structure Detection) [22] and RCNOIK algorithm (Residue Coordination Number Optimization and Improved K-means, as shown in Table 3).

**Table 3:**
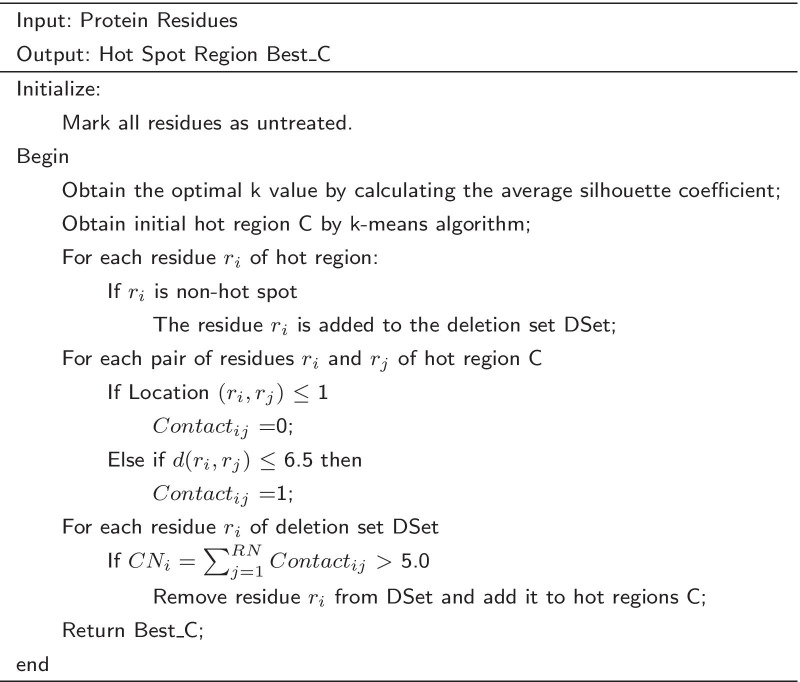
Prediction method of hot regions based on residue coordination number optimization and improved K-means(RCNOIK)

**Table 4:** LCSD algorithm based on PPRA optimization

Input: Datasets Output: Optimized Hot Region $LCSD\_Best\_CH_i$	
Step 1: Feature selection for residues in datasets;	
Step 2: A clustering-based boundary point recognition algorithm is used to obtain the predicted set of hot regions *LCSD_RH1*;	
Step 3: $LCSD\_RH1$ is optimized by PPRA to obtain hot region $LCSD\_RH2$;	
Step 4: $LCSD\_RH2$ is optimized according to the loss residue optimization strategy;	
Step 5: Repeat step 4 until there is no missing residue need to be processed;	
Step 6: Input the optimized hot regions $LCSD\_Best\_CH_i$.	

### K-means algorithm based on Silhouette coefficient

K-means algorithm [31] belongs to split clustering, which divides *n* objects into *k* clusters and makes the distance between objects within the same cluster nearest. The aim of the algorithm is to minimize the Within-Cluster Sum of Squares (WCSS). Supposing that *x* is a set of given objects, $S={S_1,S_2,\cdots ,S_k}$ represents *k* partitions, $u_i$ is the center of $S_i$, and the Within-Cluster Sum of Squares is defined as

1 $$\begin{aligned} WCSS=\sum _{i=1}^k\sum _{x\in S_i}\parallel x-u_i\parallel ^2 \end{aligned}$$

The process of k-means clustering includes five steps:

- Step 1: Input k, that is, number of groups by clustering.
- Step 2: Randomly generate k partition.
- Step 3: The center of each partition is calculated .
- Step 4: Assign the objects to the nearest clusters.
- Step 5: Repeat Step 2, Step 3 and Step 4, until WCSS converges to minimum.

The k-means algorithm is efficient method for cluster analysis in data mining, which is easy to implement. But the number of clusters k needs to be set before the algorithm carries out. An inappropriate k can produce unavailable clustering results. In order to obtain better clustering results, we used the average silhouette coefficient to determine k value that can get the best clustering result.

The effect of clustering depends on the similarity of objects within clusters and the dissimilarity between clusters. That is, the larger the distance between clusters, the better the clustering effect. And the smaller the distance between objects in the same cluster, the better the clustering result. Considering the aggregation degree of objects within clusters and the separation degree between clusters, the silhouette coefficient [32] of each object *x* is calculated as

2 $$\begin{aligned} Silhouette(x)=\frac{b(x)-a(x)}{\max \left\{ b(x),a(x)\right\} } \end{aligned}$$

where, *a*(*x*) is the average distance from object *x* to all other objects in the same cluster. And *b*(*x*) is the minimum of the average distance from object *x* to all objects of other clusters. Generally, the range of *Silhouette*(*x*) is [− 1,1]. The closer the value is to 1, the better the clustering is efficient.

### Optimization of residue coordination number

There are several distance-based measurements in the spatial structure of proteins, such as the contact number of residues, the number of neighboring residues, and the coordination number of residues. The contact residues are the basis of the interactions between protein interfaces. The neighborhood residues are important in the structure of aggregation clusters, which provide the support for constructing the protein interfaces. The coordination number of residues is the total number of neighborhood residues. Due to the hot spot residues tend to aggregate at one or more same regions in the protein interfaces, the average coordination number of hot spot residues is higher. Therefore, the average coordination number of residues can be used as the optimization condition. Two residues are supposed to contact if the distance between their $C^a$ atoms is smaller than 6.5Å. The residue’s contact is defined as

3 $$\begin{aligned} Contact_{ij}=\left\{ \begin{array}{l} 0 \qquad if \mid i-j \mid \le 1;\\ 1 \qquad if \mid i-j \mid >1 \quad and \quad d_{ij} \le 6.5\AA ;\\ \end{array} \right. \end{aligned}$$

where *d*(*i*, *j*) is the distance between two $C^a$ atoms of residue *i* and residue *j*.

4 $$\begin{aligned} d(i,j)= \sqrt{(x_j-x_i)^2+(y_j-y_i)^2+(z_j-z_i)^2} \end{aligned}$$

The coordination number of residue *i* can be defined as

5 $$\begin{aligned} CN_i= \sum _{j=1}^{RN}Contact_{ij} \end{aligned}$$

where RN is the total number of residues. We can identify hot spots according to the coordination number $CN_i$ of residues. RCNOIK algorithm (Residue Coordination Number Optimization and Improved K-means) is shown in Table 3.

### PPRA optimization

The biological properties and physical properties of protein residues are diverse, such as hydrophobicity, ASA, distribution potential, solvents and so on. Tuncbag [24] proposed that when the Pair Potentials (PP) of residues is greater than or equal to 18.0 and Relative ASA is less than or equal to 20.0, it can help to distinguish hot spot residues from protein residues. Therefore, we used these characteristics to further optimize the prediction methods and to detect the hot regions of Hub protein interaction interfaces. This optimization strategy is called PPRA (Pair Potentials and Relative ASA).

Potential energy often occurs in the space folding and the interaction interface. The potential energy of residue *i* is defined as:

6 $$\begin{aligned} PP(i)= \mid \sum _{j=1}^{N}potential(i,j)\mid \qquad if \,|i-j|\ge 4 \end{aligned}$$

where potential(*i*, *j*) denotes the potential energy of the connection between residue *i* and residue *j*.

7 $$\begin{aligned} potential(i,j)=\left\{ \begin{array}{l} contact \, potential \, of \, type(i,j) \qquad if \, d(i,j)\le 7.0;\\ \qquad \qquad 0 \qquad \qquad \qquad \qquad \qquad otherwise;\\ \end{array} \right. \end{aligned}$$

**Fig. 1:**
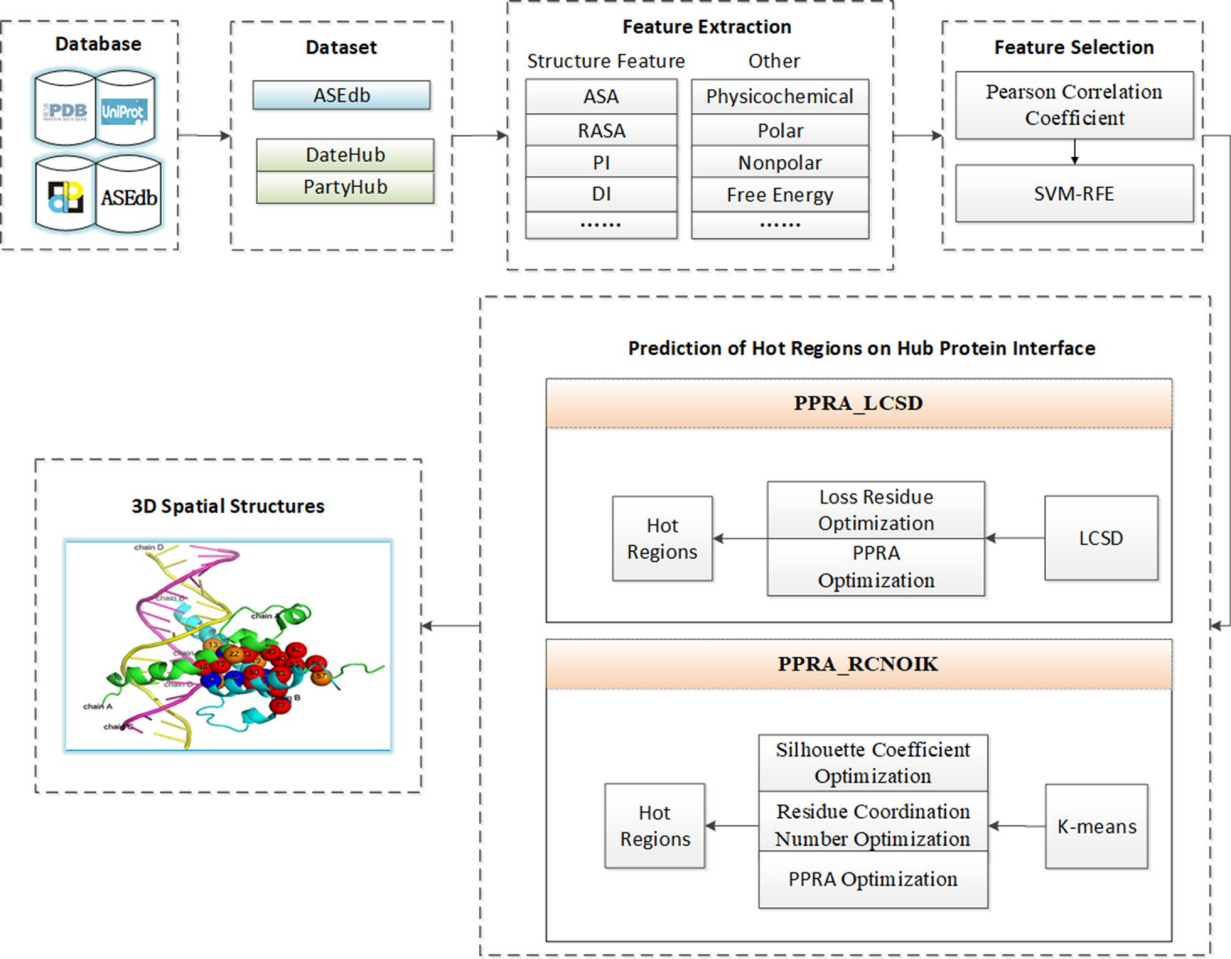
Workflow of predicting hot regions

The ASA of protein residues in complex and monomer can be calculated by PSAIA (Protein Structure and Interaction Analyzer). The Relative ASA of residue *i* in the complex is defined as:

8 $$\begin{aligned} RComASA_i=\frac{ASA \,in \, Complex_i}{maxASA_i}\times 100 \end{aligned}$$

where $maxASA_i$ represents the largest ASA of a residue in the tripeptide. Then, PPRA optimization strategy is used to optimize LCSD algorithm and RCNOIK algorithm for predicting the hot regions on Hub protein interfaces. The algorithm process is shown in Tables 4 and 5. The process of predicting hot regions is shown in the Fig. 1.

**Fig. 2:**
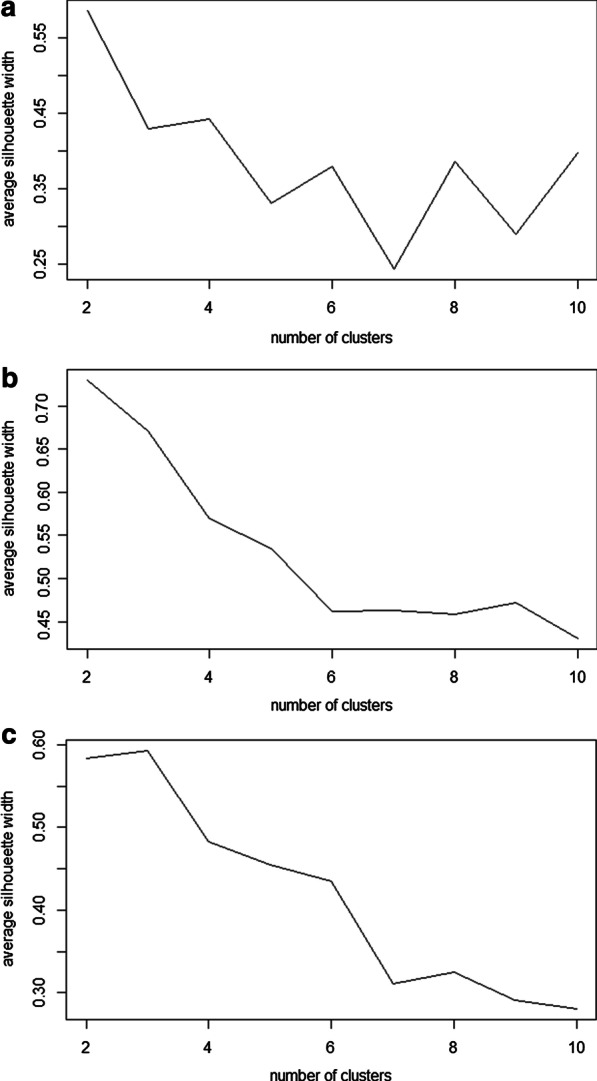
Average silhouette width graph of different k value for 1A22, 1FCC and 3HFM. **a** 1A22,k = 2 under max ASW; **b** 1FCC, k = 2 under max ASW; **c** 3HFM, k = 3 under max ASW

## Results and discussion

### Evaluation criteria

Several common measures are used to analyze the performance of prediction methods.

9 $$\begin{aligned} Precision=\frac{TP}{TP+FP} \end{aligned}$$

10 $$\begin{aligned} Recall=\frac{TP}{TP+FN} \end{aligned}$$

11 $$\begin{aligned} F1-Score=\frac{2*Precision*Recall}{Precision+Recall} \end{aligned}$$

TP (true positive): the number of correct classification in the sample of predicted positive. FP (false positive): the number of misclassification in the sample of predicted positive. FN (false negative): the number of misclassification in the sample of predicted negative.

### Experiment based on ASEdb dataset

Frist, we performed experiments used the common ASEdb dataset. Table 6 lists the results of the different methods. It can be shown that Tuncbag has very high precision, but its coverage (Recall) and F1-Score are lower. Compared with Nan and Hu, the precision (0.80) and recall (0.82) of RCNOIK are improved, which can successfully predict 80% of the real hot spot regions, and 82% of the identified hot spot regions are natural. Compared with LCSD method, RCNOIK has a slight improvement in precision and F1-Score, but its coverage is lower than LCSD method. Table 6 shows that two clustering methods LCSD and RCNOIK are effective for predicting the hot spot regions with better performance.

Next, we introduced the execution process of the RCNOIK method. First, the optimal number of clusters k can be obtained by the average silhouette width (ASW). Figure 2 gives the average silhouette width graph of different k values for protein complexes 1A22, 1FCC and 3HFM. When the average silhouette width is the largest, the value of k is the optimum. The maximum average silhouette widths of 1A22 in Fig. 2a and 1FCC in Fig. 2b can be obtained when k equals 2, and the average silhouette width of 1FCC has exceeded 0.70. The average silhouette width of 3HFM in Fig. 2c reaches the maximum when k equals 3, which is close to 0.60.

**Fig. 3:**
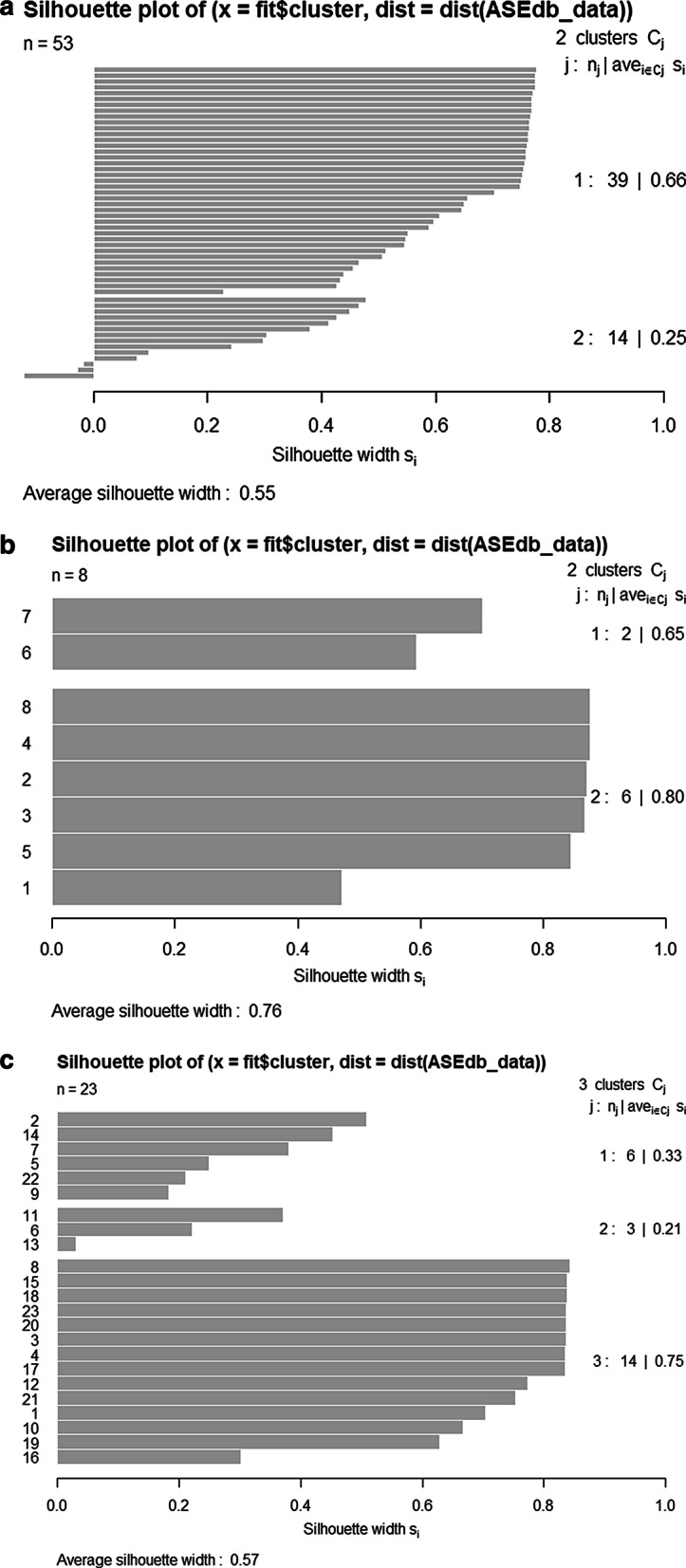
Silhouette width plots under optimized k value. **a** 1A22, Average silhouette width is 0.55 under k = 2; **b** 1FCC, Average silhouette width is 0.76 under k = 2; **c** 3HFM, Average silhouette width is 0.57 under k = 3

Figure 3 is the silhouette width plots under optimized k value of protein complex 1A22, 1FCC and 3HFM. In Fig. 3a, 1A22 has 53 residues which are clustered into two clusters. One of the clusters has 39 residues, and the other one has 14 residues. In Fig. 3b, 1FCC has 8 residues which are also clustered into two clusters. One of the clusters has 2 residues, and the other one has 6 residues. In Fig. 3c, 3HFM has 23 residues which are clustered into three clusters, including 6 residues, 3 residues and 14 residues respectively.

**Fig. 4:**
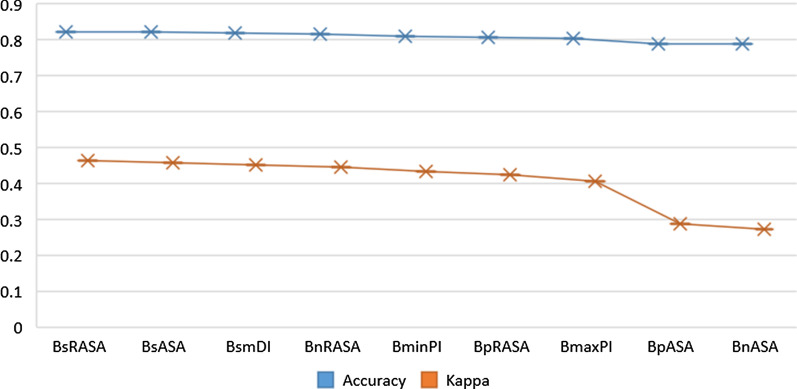
Optimum feature combination of clustering

**Fig. 5:**
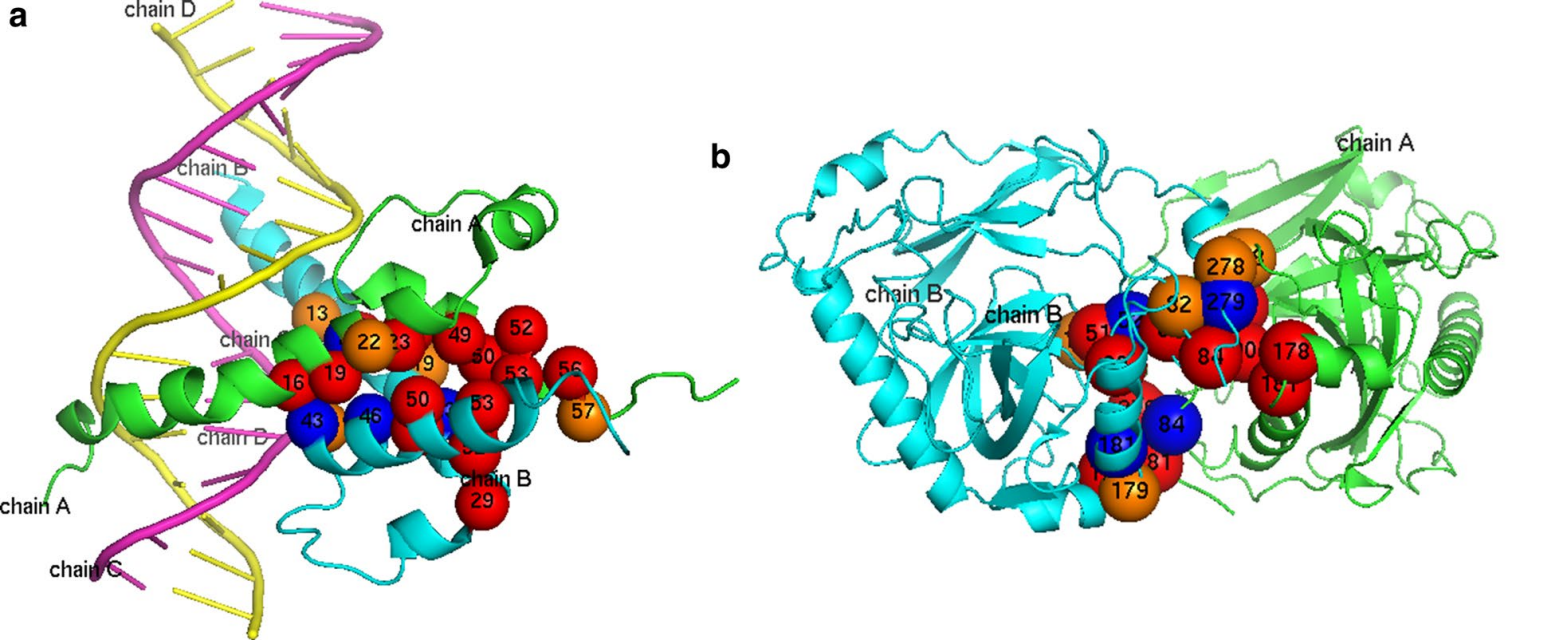
Three-dimensional spatial structures of DateHub proteins 1A0A and 1E9G. **a** 1A0A; **b** 1E9G

In the experiments, we found that the different feature combinations have great influence on clustering results. The optimum feature combination are composed of nine features, namely: BsRASA, BsASA, BsmDI, BnRASA, BminPI, BpRASA, BmaxPI, BpASA and BnASA. The accuracy and kappa coefficient of these nine features are shown in Fig. 4. Kappa coefficient [33, 34] is an index to measure the accuracy of classification,which is defined as

12 $$\begin{aligned} kappa=\frac{p_0-p_e}{1-p_0} \end{aligned}$$

where $p_0$ is total classification accuracy. Supposing that $a_i$ and $b_i$ are true samples and predicted samples respectively, $p_e$ can be defined as

13 $$\begin{aligned} p_e=\frac{\sum _{i=1}^c{a_i\times b_i}}{n \times n} \end{aligned}$$

**Table 5:** RCNOIK algorithm based on PPRA optimization

Input: Datasets Output: Optimized Hot Region $RCNOIK\_Best\_CH_i$	
Step 1: Feature selection for residues in datasets;	
Step 2: Calculate the sum of distance squares of residues and the average silhouette value to obtain the optimal k value;	
Step 3: K-means is used to obtain the hot regions *RCNOIK_RH1*;	
Step 4: $RCNOIK\_RH1$ is optimized by PPRA to obtain hot region $RCNOIK\_RH2$;	
Step 5: $RCNOIK\_RH2$ is optimized according the RCNO strategy;	
Step 6: Input the optimized hot regions $RCNOIK\_Best\_CH_i$.

**Table 6:** Performance of predicting hot regions by different methods

Method	Precision	Recall	F1-Score
Tuncbag	1	0.20	0.33
Nan	0.67	0.40	0.49
Hu	0.78	0.70	0.74
LCSD	0.78	0.83	0.80
RCNOIK	0.80	0.82	0.83

### Experiment based on hub protein datasets

Two clustering methods LCSD and RCNOIK were also carried out with DateHub and PartyHub datasets. Table 7 shows the results of LCSD and RCNOIK methods for hot region prediction on DateHub and PartyHub datasets. Using the same evaluation criteria as previous studies, the performance of the two methods on two datasets were evaluated. For the results, the coverages of two methods on DateHub dataset are higher than those on PartyHub dataset, which coincides with the fact that DateHub is likely to have more hot spots. In addition, although LCSD and RCNOIK have the same coverage on both datasets, RCNOIK is slightly higher than LCSD in Precision. RCNOIK has the highest Precision (0.64) on DateHub dataset, and the balance performance F1-Score is also better. And RCNOIK can successfully predict 54% of natural hot regions on the DateHub dataset, and 64% of identified hot regions are natural hot regions.

To further improve the performance of prediction results, we optimized the prediction methods by using PPRA. Table 8 lists the prediction performance based on PPRA optimization, and shows that two optimized methods have been improved under Precision, Recall and F1-Score. PPRA_LCSD and PPRA_RCNOIK methods improve the accuracy on both datasets. Moreover, the hot spots predicted on the DateHub dataset are more than those on the PartyHub dataset, and the coverages of both methods are increased to 70%. PPRA_RCNOIK has the best prediction effect on DataHub dataset ($F1-Score=0.78$).

To further analyze the predicted performance, Tables 9 and 10 list detailed prediction results of protein complexes (1A0A and 1E9G) of DataHub dataset based on RCNOIK and PPRA_RCNOIK. For Table 9, many hot spots of hot regions are lost, and more false hot spots are predicted to be in the hot regions. For Table 10, some of the lost hot spots are recovered by PPRA optimization, and more non-hot spots can be removed. In addition, the results of clustering methods used in this paper are closer to those of classification methods (The classification results are shown in Table 11), which can get most of the same hot regions. Further, the optimized clustering methods used are effective for predicting the hot regions of Hub protein interaction interfaces.

To observe predicted hot regions more intuitively, the three-dimensional spatial structures of 1A0A and 1E9G are given. As shown in Fig. 5, the different colors represent different chains and different shapes represent different types of chains. The complex 1A0A in Fig. 5a consists of four chains, A, B, C and D, in which A and B chains are main chains, C and D chains are side chains. Hot regions appear in A and B chains. The red spheres are the predicted hot spots, which are the same as the natural hot spots. After optimization, 15 hot spots are predicted. Blue spheres are the natural hot spots, but they are judged to be non-hot spots. We can see that four natural hot spots (A20, B23, B43, B46) in the hot regions are misjudged as non-hot spots. Orange spheres represent the predicted false hot spots, and five false hot spots (A22, A57, B13, B19, B42) appear in the predicted hot region structure. The complex 1E9G in Fig. 5b consists of two main chains A and B, without side chains. Four natural hot spots (A84, B52, B181, B279) are misjudged as non-hot spots, and five non-hot spots (A128, B82, B128, B179, B278) are predicted as hot spots. Two three-dimensional spatial structures show that most of the hot regions of Hub protein can be correctly predicted.

**Table 7:** Prediction performance of two methods on DateHub and PartyHub (before optimization)

Dataset	Method	Precision	Recall	F1-Score
DateHub	LCSD	0.58	0.54	0.56
	RCNOIK	0.64	0.54	0.59
PartyHub	LCSD	0.48	0.51	0.49
	RCNOIK	0.51	0.51	0.51

**Table 8:** Prediction performance based on PPRA optimization (after optimization)

Dataset	Method	Precision	Recall	F1-Score
DateHub	PPRA_LCSD	0.78	0.70	0.74
	PPRA_RCNOIK	0.89	0.70	0.78
PartyHub	PPRA_LCSD	0.73	0.62	0.67
	PPRA_RCNOIK	0.89	0.62	0.73

**Table 9:** Prediction results of 1A0A and 1E9G by clustering based on RCNOIK

PDB ID	Natural hot spots predicted	Natural hot spots unpredicted	False hot spots predicted
1A0A	A16, A19, A49, A52, A53, B16, B29, B52, B53	A20, A23, A46, A50, A56, B23, B43, B46, B49, B50	A22, A43, A47, A57, B13, B19, B22, B28, B42, B47, B54, B57
1E9G	A51, A52, A90, A279, A281, B51, B90	A84, A87, A178, A181, B52, B84, B87, B178, B181, B279	A82, A126, A127, A128, A184, A278, B82, B16, B128, B179, B180, B278, B281, B283

**Table 10:** Prediction results of 1A0A and 1E9G by PPRA optimization

PDB ID	Hot spots recovered	Hot spots unrecovered	False hot spots recovered	False hot spots
1A0A	A23, A46, A50, A56, B49, B50	A20, B23, B43, B46	A43, A47, B22, B28, B47, B54, B57	A22, A57, B13, B19, B42
1E9G	A87, A178, A181, B84, B87, B178	A84, B52, B181, B279	A82, A126, A127, A184, A278, B16, B180, B281, B283	A128, B82, B128, B179, B278

**Table 11:** Prediction results of 1A0A and 1E9G by classification methods

PDB ID	Method	Natural hot spots predicted	Natural hot spots unpredicted	False hot spots
1A0A	Boosting	A16, A19, A49, A53, A56, B16, B29, B49, B50, B52, B53	A20, A23, A46, A50, A52, B23, B43, B46	A15, A22, A57, B13, B19, B22, B42
	Gradient boosting	A16, A19, A23, A46, A49, A52, A53, B16, B29, B49, B52, B53	A50, B23, B43, B46	A22, A26, B13, B19, B42, B57
	Random forest	A16, A19, A23, A46, A49, A50, A52, A53, B16, B29, B43, B49, B52, B50, B53, A56	A20, B23, B46	A22, A57
1E9G	Boosting	A51, A52, A90, A279, A281, B51, A178, A181, B84, B90	A87, A84, B52, B87, B178, B181, B279	A82, A126, A128, B82, B128, B179
	Gradient boosting	A51, A52, A87, A90, A279, A281, B51, B52, A178, A181, B84, B178,	A84, B87, B90, B181, B279	A126, A128, B82, B126, B128, B278
	Random forest	A51, A52, A87, A90, A279, A281, B51, A178, A181, B84, B87, B90, B279	A84, B52, B178, B181	A128, B179

## Conclusions

In PPIN, a minority of Hub proteins have many interacting partners. Obviously, the connectivity of proteins is related to protein function. However, the characteristics of Hub protein have not been fully understood. There are still many important and meaningful problems need to be solved about Hub protein interaction interfaces. In this paper, two clustering methods were optimized to predict hot regions on Hub protein interaction interfaces. First, LCSD method and RCNOIK method were used to identify the hot region structures of Hub protein interaction interfaces. Then, PPRA optimization strategy was used to optimized the predicted hot regions. The experimental results show that the improved methods are effective in predicting hot regions of Hub protein interaction interfaces, and have better performance than other methods. In addition, compared with the classification results, the clustering method adopted can obtain essentially identical results with those of classification methods, which further illustrates that the improved methods are feasible for predicting hot regions.

Combining with the previous works, the following aspects need to be further explored. We will continue to study the characteristics of different Hub protein interfaces and explore which types of Hub protein interface are more likely to form hot region structures. In addition, the structural domain can reflect the mechanism of PPIs, which is helpful to understanding the cell function. Next, we will also continue to study the function of domain on Hub protein interaction interfaces, and analyze the importance of Hub protein for predicting drug-target interactions.

## Data Availability

The dataset for constructing model is available in the ASEdb database (http://nic.ucsf.edu/asedb/). The solvent accessible surface area (ASA) and relative accessible surface area (RASA) of Hub protein are available at HotPoint server (http://prism.ccbb.ku.edu.tr/hotpoint/).The hub protein complexes are available in the protein data bank (http://www.rcsb.org/).

## Abbreviations

PPIs: Protein–protein interactions
PPIN: Protein–protein interaction network
PPRA: Pair potentials and relative ASA
WCSS: Within-cluster sum of squares
LCSD: Local community structure detection
RCNOIK: Residue coordination number optimization and improved K-means
ASA: accessible surface area
RASA: Relative accessible surface area
TP: True positive
FP: False positive
FN: False negative

## References

[1] Qian EA, Han Y, Messina MS, Maynard HD, Kral P, Spokoyny AM (2019). Multivalent cluster nanomolecules for inhibiting protein–protein interactions. Bioconjug Chem.

[2] Scott DE, Bayly AR, Abell C, Skidmore J (2016). Small molecules, big targets: drug discovery faces the protein–protein interaction challenge. Nat Rev Drug Discov.

[3] Deng SP, Zhu L, Huang DS (2016). Predicting hub genes associated with cervical cancer through gene co-expression networks. IEEE/ACM Trans Comput Biol Bioinform.

[4] Zhu L, Guo W, Deng SP, Huang DS (2016). Chip-pit: enhancing the analysis of chip-seq data using convex-relaxed pair-wise interaction tensor decomposition. IEEE/ACM Trans Comput Biol Bioinf.

[5] Cukuroglu E, Engin HB, Gursoy A, Keskin O (2014). Hot spots in protein–protein interfaces: towards drug discovery. Prog Biophys Mol Biol.

[6] Lin XL, Zhang XL, Zhou FL (2014). Protein structure prediction with local adjust tabu search algorithm. BMC Bioinform.

[7] Bandyopadhyay S, Mallick K (2017). A new feature vector based on gene ontology terms for protein–protein interaction prediction. IEEE/ACM Trans Comput Biol Bioinform.

[8] Keskin O, Tuncbag N, Gursoy A (2016). Predicting protein–protein interactions from the molecular to the proteome level. Chem Rev.

[9] Bhardwaj J, Gangwar I, Panzade G, Shankar R, Yadav SK (2016). Global de novo protein–protein interactome elucidates interactions of drought-responsive proteins in horse gram (*Macrotyloma uniflorum*). J Proteome Res.

[10] Luck K, Kim DK, Calderwood MA (2020). A reference map of the human binary protein interactome. Nature.

[11] Jeong H, Mason SP, Barabasi AL (2001). Lethality and centrality in protein networks. Nature.

[12] Mondragon RJ (2020). Estimating degree-degree correlation and network cores from the connectivity of high-degree nodes in complex networks. Sci Rep.

[13] Aper SJA, Hamer AD, Wouters SFA, Lemmens LJM, Ottmann C, Brunsveld L, Merkx M (2018). Protease-activatable scaffold proteins as versatile molecular hubs in synthetic signaling networks. ACS Synth Biol.

[14] Dayebgadoh G, Sardiu ME, Florens L, Washburn MP (2019). Biochemical reduction of the topology of the diverse WDR76 protein interactome. J Proteome Res.

[15] Deng SP, Lin Z, Huang DS (2015). Mining the bladder cancer-associated genes by an integrated strategy for the construction and analysis of differential co-expression networks. BMC Genom.

[16] Yu H, Kim PM, Sprecher E (2007). The importance of bottlenecks in protein networks: correlation with gene essentiality and expression dynamics. PLoS Comput Biol.

[17] Hahn MW, Kern AD (2005). Comparative genomics of centrality and essentiality in three eukaryotic protein-interaction networks. Mol Biol Evol.

[18] Batada N, Hurst LD, Tyers M (2006). Evolutionary and physiological importance of hub proteins. PLoS Comput Biol.

[19] He X, Zhang J (2006). Why do hubs tend to be essential in protein networks?. PLoS Genet.

[20] Xia JF, Yue Z, Di Y, Zhu X, Zheng CH (2016). Predicting hot spots in protein interfaces based on protrusion index, pseudo hydrophobicity and electronion interaction pseudopotential features. Oncotarget.

[21] Zhang XL, Lin XL, Zhao JF, Hang QQ, Xu X (2019). Efficiently predicting hot spots in PPIs by combining random forest and synthetic minority over-sampling technique. IEEE/ACM Trans Comput Biol Bioinform.

[22] Lin XL, Zhang XL (2018). Prediction of hot regions in PPIs based on improved local community structure detecting. IEEE/ACM Trans Comput Biol Bioinform.

[23] Cukuroglu E, Gursoy A, Keskin O (2010). Analysis of hot region organization in hub proteins. Ann Biomed Eng.

[24] Tuncbag N, Gursoy A, Keskin O (2009). Identification of computational hot spots in protein interfaces: combining solvent accessibility and inter-residue potentials improves the accuracy. Bioinformatics.

[25] Hu J, Zhang XL, Liu XM, Tang JS (2015). Prediction of hot regions in protein–protein interaction by combining density-based incremental clustering with feature-based classification. Comput Biol Med.

[26] Nan, D.F., Zhang, X.L.: Prediction of hot regions in protein–protein interactions based on complex network and community detection. In: Proceedings of IEEE International Conference on Bioinformatics and Biomedicine, 18–21 December 2013, ShangHai, pp. 17–23 (2013)

[27] Ekman D, Light S, Bjorklund AK (2006). What properties characterize the hub proteins of the protein–protein interaction network of *Saccharomyces cerevisiae*?. Genome Biol.

[28] Lin XL, Zhang XL, Xu X (2019). Efficient classification of hot spots and hub protein interfaces by recursive feature elimination and gradient boosting. IEEE/ACM Trans Comput Biol Bioinform.

[29] Yu H, He F, Pan Y (2018). A novel region-based active contour model via local patch similarity measure for image segmentation. Multimed Tools Appl.

[30] Choobdar S, Ahsen ME, Crawford J, Tomasoni M, Fang T (2019). Assessment of network module identification across complex diseases. Nat Methods.

[31] Jeong YJ, Lee J, Moon J, Shin JH, Lu WD (2018). K-means data clustering with memristor networks. Nano Lett.

[32] Barth C, Becker C (2018). Machine learning classification for field distributions of photonic modes. Commun Phys.

[33] Tang W, Hu J, Zhang H, Wu P, He H (2015). Kappa coefficient: a popular measure of rater agreement. Shanghai Arch Psychiatry.

[34] Zec S, Soriani N, Comoretto R, Baldi I (2017). High agreement and high prevalence: the paradox of Cohen’s kappa. Open Nurs J.

